# Exploring Blood Flow Restriction Exercise Protocols for Elderly Populations: A Scoping Review of Cuff Pressure, Frequency, and Duration for Muscle Strength, Hypertrophy, and Functional Abilities Outcomes

**DOI:** 10.3390/jcm14124185

**Published:** 2025-06-12

**Authors:** Mohamed Abdelaziz Emam, Ahmed Elsayed, Tibor Hortobágyi, Wafaa Mahmoud Amin, Shazia Malik, Olfat Ibrahim Ali

**Affiliations:** 1 János Szentágothai Neurosciences Division, Semmelweis University, 1085 Budapest, Hungary; 2 Basic Sciences Department, Faculty of Physical Therapy, Kafr Elsheikh University, Kafr Elsheikh 33511, Egypt; 3 Department of Physiotherapy, Faculty of Health Sciences, Semmelweis University, 1085 Budapest, Hungary; ahmedashraaf716@gmail.com; 4 Department of Kinesiology, Hungarian University of Sports Science, 1123 Budapest, Hungary; t.hortobagyi@umcg.nl; 5 Department of Sport Biology, Institute of Sport Sciences and Physical Education, University of Pécs, 7622 Pécs, Hungary; 6 Center for Human Movement Sciences, University of Groningen, 9713 AV Groningen, The Netherlands; 7 College of Nursing and Health Sciences, Jazan University, Jazan 45142, Saudi Arabia; wafaa_770@yahoo.com (W.M.A.); haz_physio@yahoo.com (S.M.); 8 Department of Basic Science for Physical Therapy, Faculty of Physical Therapy, Cairo University, Giza 12613, Egypt; 9 Physical Therapy Program, Batterjee Medical College, Jeddah 21442, Saudi Arabia

**Keywords:** blood flow restriction training, older adults, muscle strength, hypertrophy, functional capacity, scoping review, exercise prescription

## Abstract

**Background/Objectives:** As aging leads to a decline in muscle mass, strength, and functional capacity, identifying effective, low-risk interventions for older adults is essential. Blood flow restriction training (BFRT) has gained recognition as a potential substitute for traditional high-load resistance training, offering comparable benefits with reduced mechanical stress. This scoping review explores current BFRT protocols—specifically cuff pressure, training frequency, and duration—and their impact on muscular strength, hypertrophy, and functional capabilities among healthy elderly individuals. **Methods:** Following PRISMA-ScR and Arksey and O’Malley’s framework, six databases were searched (2010–2024), yielding 13 eligible studies. Data were charted for BFRT parameters, training regimens, and outcomes related to strength, muscle size, and functionality. Risk of bias was assessed using Cochrane guidelines. **Results:** Low-load BFRT (20–40% 1RM), applied 2–4 times weekly for 6–12 weeks, significantly improved muscle strength, hypertrophy (e.g., quadriceps CSA), and functional performance (e.g., TUG, 6MWT). Cuff pressures ranged from 50 to 80% arterial occlusion pressure (AOP) for the lower limbs and 30–50% above systolic pressure for the upper limbs. Wider cuffs enhanced safety and comfort. BFRT demonstrated comparable or superior outcomes to conventional training in most studies, with minimal adverse effects reported. **Conclusions:** The existing evidence suggests that BFRT may be a promising intervention for improving muscle health and functionality in older adults; however, future research should focus on standardizing protocols, long-term outcomes, and tailored guidelines to optimize safety and efficacy.

## 1. Introduction

Aging is associated with a number of physiological changes that can contribute to declines in muscle strength and power, as well as overall functional performance [1]. These impairments reduce independence and increase the risk of falls and other health complications. As individuals grow older, skeletal musculature diminishes approximately 3–8% every ten years following age 30 [2]. The simultaneous decline in both muscle volume and force production, known as sarcopenia [3], carries significant functional and metabolic implications, including elevated risks of falling and mortality [4,5].

Considering demographic changes, particularly in Western societies [6], it is increasingly important to identify suitable and evidence-based strategies that combat age-related functional deterioration. Resistance training has been extensively acknowledged as a beneficial intervention to counteract these age-related changes and improve functional abilities in older adults [7].

However, traditional high-intensity resistance training may be inappropriate or impractical for many seniors, particularly those with pre-existing health conditions or mobility limitations. Individuals also vary in their capacity to tolerate the intense mechanical stress imposed on their joints during vigorous resistance exercise [8]. Blood flow restriction training (BFRT) has gained recognition as an innovative methodology that could offer a feasible substitute for standard resistance exercises among elderly populations [9,10,11,12]. In these techniques, blood flow restriction (BFR) is achieved by utilizing pneumatic wraps to apply external compression of underlying tissues. This pressure is sufficient to compress superficial veins (low-pressure vessels) but not arteries (high-pressure vessels). This creates a partial occlusion: venous blood cannot return to the heart, causing metabolite accumulation (e.g., lactate), while arterial blood continues supplying oxygen. This imbalance induces metabolic stress, triggering muscle hypertrophy and strength adaptations akin to high-load training [13].

This methodology typically integrates partial vascular occlusion to working muscle groups through proximal positioning of pneumatic wraps with moderate-to-low intensity (20–40% of one-repetition maximum (1RM)) strength exercises of the targeted limb [14]. BFR exercise stimulates increased physiological metabolic demands, which includes increased growth hormone secretion and increased activation of fast-twitch muscle fibers, generating ischemia in the limbs [15,16].

A scoping review was chosen over a systematic review or meta-analysis due to the heterogeneity of BFRT protocols (e.g., differences in cuff pressures, frequencies, durations, and exercise modalities). The limited number of high-quality RCTs would not allow valid pooling of effect sizes. Following Arksey and O’Malley’s framework [17], the objective was to map the key concepts, identify research gaps, and summarize trends in BFRT protocols applied to older adults, rather than to evaluate efficacy statistically. A scoping review was chosen over a systematic review or meta-analysis due to the heterogeneity of BFRT protocols (e.g., differences in cuff pressures, frequencies, durations, and exercise modalities). The limited number of high-quality RCTs that would not allow valid pooling of effect sizes. Following Arksey and O’Malley’s framework, the objective was to map the key concepts, identify research gaps, and summarize trends in BFRT protocols applied to older adults, rather than to evaluate efficacy statistically.

The findings from this scoping review will enhance the likelihood of practical clinical adoption of blood flow restriction training, thereby allowing healthcare professionals to implement suitable parameters (e.g., cuff parameters, number of sets, repetition schemes and duration) for BFRT interventions protocols in peer-reviewed literature. In addition, based on our assessment of prior studies, the study will recommend future directions for blood flow restriction interventions targeting healthy elderly populations, along with appropriate guidelines for exercise documentation.

Therefore, this scoping review explores current evidence on blood flow restriction training protocols, focusing on cuff pressure, training frequency, and duration, and their impact on muscular strength, hypertrophy, and functional capabilities in healthy elderly populations.

## 2. Objectives

This scoping study will be guided by the following inquiries regarding particular components of the intervention: What are the variations in blood flow restriction protocols in terms of cuff pressure, frequency, duration, and how do they impact muscular strength, hypertrophy, and functional capabilities in healthy elderly individuals?

## 3. Methods

### 3.1. Search Strategy

Scoping reviews are recommended for mapping key principles, knowledge gaps, and research methodologies within specific fields. They can additionally inform subsequent investigations and potential future systematic reviews on the subject. As the research questions were exploratory in nature, the present study was conducted as a scoping review following the methodology outlined by Arksey and O’Malley [17]. This study also adheres to the Preferred Reporting Items for Systematic Reviews and Meta-analysis Extension for Mapping Reviews (PRISMA-ScR) protocols; these were developed by the EQUATOR (Enhancing the Quality and Transparency Of health Research) Network for the development of reporting guidelines, and subsequently approved by the St. Michael’s Hospital Research Ethics Board [18].

The following electronic databases were queried for publications spanning from 2010 to December 2024: PubMed, Web of Science (WOS), Scopus, CINAHL, ProQuest, and CEN-TRAL. Database searches were executed without limitations (“All field/All text” queries), with the exception of Scopus where investigations were confined to “Title, Abstract, Keywords” fields.

The search was performed separately by the two researchers using the following search string for all databases: (“blood flow restriction” OR “occlusion training” OR “vascular occlusion” OR KAATSU OR “ischemi* training”) AND (old* OR elder* OR sarcopeni* OR “musc* atrophy”) AND (“optimal dose” OR protocol OR parameters OR intensity OR frequency OR “cuff pressure” OR duration).

**Figure 1:**
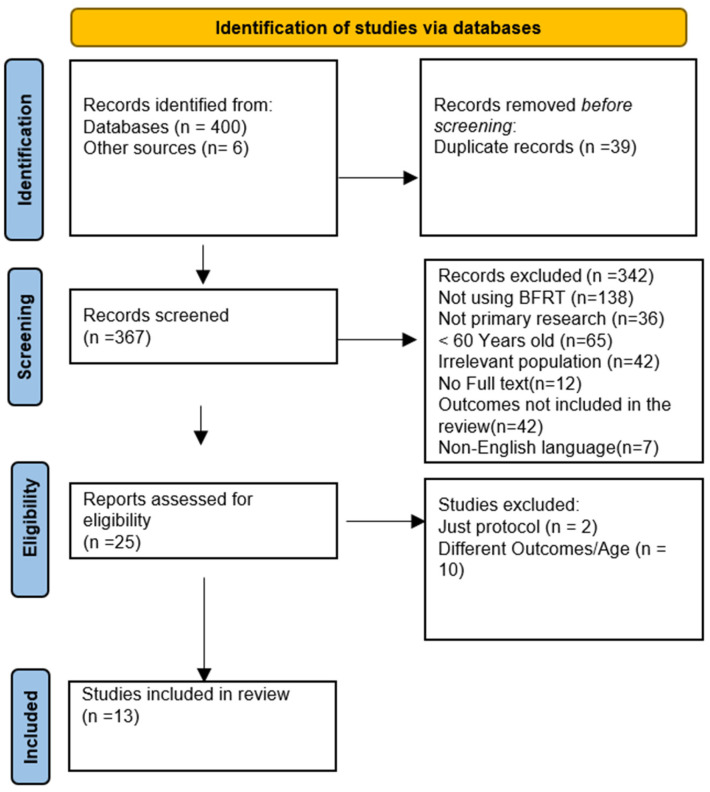
Flow diagram for new systematic reviews using PRISMA 2020, which included database searches.

Study information, encompassing title and abstract, were extracted from the databases and transferred to Rayyan.ai software (version accessed in 2024). Prior to additional analysis of the manuscripts, all duplicates were eliminated (for search process see Figure 1).

### 3.2. Eligibility Criteria

All studies underwent screening and eligibility evaluation according to our selection and rejection criteria, which were derived from the PICOS framework (specifically extracting participant demographics, treatment protocols, comparative interventions, outcome measures, and research design information).

Manuscripts were deemed relevant if (1) participants were seniors aged 60 years or above. (2) Intervention was BFR training using various cuff pressures, frequencies, or durations. (3) Studies reported outcomes related to muscle strength, hypertrophy, or functional activities. (4) Research design was controlled trials, randomized controlled trials, and cohort studies. (5) Studies published in English and peer-reviewed articles. Manuscripts were excluded from consideration if (1) subjects had received a substance previously demonstrated to enhance muscular gains or (2) the manuscript was composed in a language other than English. (3) Review articles or opinion pieces.

This review focused exclusively on healthy older adults to reduce confounding variables associated with comorbidities such as osteoarthritis, diabetes, or cardiovascular disease, which could independently affect muscle function, training tolerance, and safety outcomes. By limiting our scope to healthy elderly populations, we aimed to isolate the effects of blood flow restriction training parameters on strength, hypertrophy, and function without the influence of disease-specific adaptations or limitations. Future reviews may consider clinical populations to guide condition-specific protocols.

### 3.3. Data Extraction and Charting

Two independent researchers extracted data from the studies included in this scoping review. The data extraction captured details regarding the population, concept, context, study methodologies, and key results related to the review questions. Additionally, information on the research design, target population, sample size, procedures, BFRT intervention specifics, exercise regimens, and outcome measures was documented.

The blood flow restriction interventions were described in terms of type, dosage, cuff settings, and methods for progressing or modifying the training stimulus. In cases where intervention outcomes were evaluated at several intervals, the terminal post- intervention assessment point was exclusively incorporated.

A data extraction template was collaboratively established by two investigators to direct information collection. Both reviewers independently recorded the information, compared their observations, and progressively enhanced the extraction template through an ongoing refinement process. Conflicts regarding publication inclusion were addressed through consultation with an additional reviewer. The collected information from the selected publications is displayed in Tables S1–S4 (see Supplementary Materials).

### 3.4. Risk of Bias

**Figure 2:**
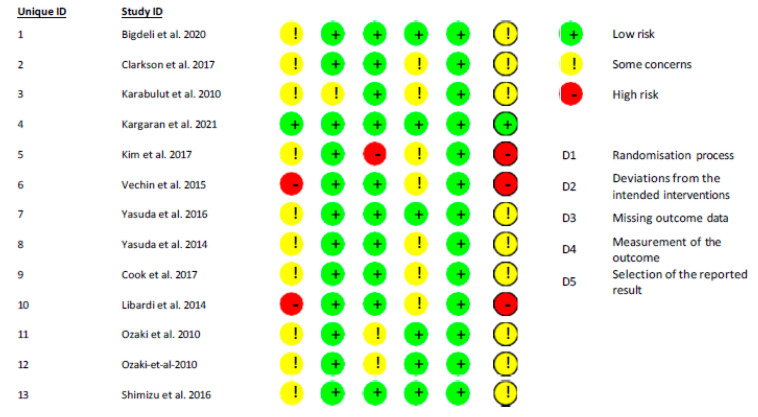
Risk of bias summary for included studies [9,19,20,21,22,23,24,25,26,27,28,29,30].

In accordance with guidelines from the Cochrane Handbook for Systematic Reviews of Interventions, bias potential was evaluated using six parameters that were separately assessed for each investigation. Within this framework, selection bias, performance bias, detection bias, as well as participant withdrawal and selective reporting issues were examined by the reviewers Figure 2.

### 3.5. Synthesis of Results

Percentage changes [((MEANpost − MEANpre)/MEANpre) × 100] of muscular strength and muscle mass were computed for each study. When multiple evaluation techniques were employed, the lowest and highest average values from each methodology were documented (Tables S1–S3).

## 4. Results

From 400 manuscripts identified through systematic literature search, thirteen met the inclusion criteria and were included in the review, as illustrated by the PRISMA flowchart (Figure 1). The thirteen selected studies included eight RCTs focus on low resistance training [9,19,20,21,22,23,24,25], three RCTs focus on walking training [26,27,28], and two RTCs focusing on functional training [29,30].

### 4.1. Included Study Characteristics

Table S2 presents the extracted information along with a narrative synthesis. Some studies compared the effectiveness of BFRT with other types of training on muscular strength, muscle size, and functional training in healthy older adults. For example, five RCTs compared the impacts of low-intensity protocols with BFRT and high resistance training [9,19,20,21,24]. Another study compared the impacts of low-intensity exercise with BFRT and medium resistance training [22].One RCT compared the impacts of low-intensity exercise with and without BFRT [25]. Another study compared the impacts of low-intensity exercise with BFRT and the control group with sedentary lifestyle [23]. One study examined the effects of functional training with and without BFRT [29]. Another study examined the effects of dual task training with and without BFRT [30]. An additional three studies examined the effects of walking training with and without BFRT [26,27,28]. Across most studies, resistance protocols incorporated leg press, knee flexion, and extension exercises. Depending on the research design, the BFRT interventions were implemented bi-weekly, tri-weekly, or quad-weekly, with program durations spanning from four to twelve weeks.

### 4.2. BFRT Parameters/Cuff Parameters

Blood flow restriction training has gained attention for its potential to enhance muscular strength, hypertrophy, and functional performance, particularly in older populations. Based on the reviewed studies, key parameters for blood flow restriction training can be standardized to improve reproducibility and efficacy in future research (Table S1).

#### 4.2.1. Cuff Placement and Pressure

The placement of the cuffs is critical for effective blood flow restriction. For lower body exercises, cuffs are typically positioned proximally on the thighs, while for upper body exercises, they are applied to the upper arms. The pressure applied varies based on the targeted region and participant characteristics. A commonly recommended approach is to set the cuff pressure as a percentage of the arterial occlusion pressure (AOP), typically starting at 40–50% AOP for the lower body and 30–40% AOP for the upper body. In studies that use absolute pressures instead, lower limb pressures generally range between 150 and 200 mmHg, while upper limb pressures fall between 100 and 130 mmHg. However, these absolute values are variable and may not align directly with individualized AOP or systolic blood pressure. Therefore, individualized pressure determination (e.g., using Doppler ultrasound or limb occlusion testing) is increasingly preferred to ensure safety and efficacy. In progressive protocols, pressure may be increased incrementally by 10–20 mmHg per session or weekly, reaching higher values such as 200–270 mmHg, depending on tolerance and study design.

#### 4.2.2. Cuff Characteristics and Systems

The width of the cuffs plays a role in determining the required pressure and participant comfort. Studies utilized cuffs with widths ranging from 5 cm (narrow cuffs) to 18 cm (wider cuffs). Elastic and pneumatic systems, such as the KAATSU Master, are frequently employed to ensure precise pressure adjustments.

#### 4.2.3. Exercise Intensity and Training Protocol

Exercise intensity in BFRT protocols often involves low-load resistance training (LL-BFR) set at 20–50% of one-repetition maximum (1RM). For walking-based protocols, intensity is set at approximately 45% of heart rate reserve or a comfortable walking speed of around 4 km/h. Training frequency generally ranges from two to four sessions per week, with interventions lasting six to twelve weeks. During each session, cuffs remain inflated for 10–20 min, providing sufficient time to induce metabolic stress while ensuring safety.

#### 4.2.4. Monitoring and Safety

Rate of perceived exertion (RPE) is commonly used to monitor training intensity and adjust cuff pressures dynamically. To prioritize safety, cuffs are deflated between sets for rest periods of 1–5 min and immediately post-exercise to restore normal blood flow. This approach minimizes the risk of adverse effects, especially in vulnerable populations such as older adults.

Some studies reported cuff pressures as high as 270 mmHg, particularly when using narrow or elastic cuffs. However, these protocols typically involved gradual progression, individualized pressure adjustment, and careful monitoring of perceived exertion and discomfort. Importantly, no adverse events were reported in studies using these higher pressures. These findings support the need for standardizing safe pressure thresholds tailored to limb size, cuff width, and participant health status.

### 4.3. Outcome Measures

The included studies used a range of outcome measures. These included muscle strength, hypertrophy especially cross-sectional area (CSA) of quadriceps muscle, and functional ability; they also evaluated 30-s Stand-Up Test (N), Timed Up and Go test (TUG), Sharpened Romberg Test, 6-min Walking Test (m), instrumented leg press, leg extension 1RM. Additionally, they also conducted MRI scan for muscle size (Tables S2 and S3).

### 4.4. Outcomes

The reviewed studies collectively affirm the efficacy of blood flow restriction training for improving muscular strength, hypertrophy, and functional performance, particularly in elderly individuals. Key findings are summarized as follows: effectiveness in strength, hypertrophy, and function (Table S3).

1. Muscle Strength: BFRT consistently results in significant strength gains across various training modalities. For example, Bigdeli et al. (2020) [29] and Clarkson et al. (2017) [26] demonstrated substantial improvements in physical strength metrics compared to sedentary or conventional training groups. These outcomes underline BFRT’s capacity to enhance muscular performance effectively.
2. Muscle Hypertrophy: Several studies, such as those by Libardi et al. (2015) [9] and Cook et al. (2017) [24], reported increased muscle cross-sectional area, highlighting the hypertrophic benefits of blood flow restriction training. This makes it a viable strategy for muscle mass preservation and growth, especially in populations at risk of muscle atrophy.
3. Functional Performance: Blood flow restriction training has been shown to improve functional abilities, such as walking endurance and balance. For instance, Kargaran et al. (2021) [30] and Yasuda et al. (2014) [23] observed significant gains in mobility and daily functional tasks, demonstrating the versatility of BFRT in addressing real-world functional limitations.

#### 4.4.1. Comparisons of Conventional and High-Load Training

1. Comparable Results at Lower Intensity: Blood flow restriction training provides comparable strength and hypertrophy improvements to high-load resistance training but with significantly lower loads. Studies like those by Libardi et al. (2015) [9] and Vechin et al. (2015) [21] demonstrated similar muscle and strength outcomes between BFRT and HRT, supporting BFRT as a safer, low-load alternative for populations unable to perform heavy lifting.
2. Unique Functional Benefits: Blood flow restriction training often exceeds control groups in functional outcomes, as highlighted in studies like those by Clarkson et al. (2017) [26] and Kargaran et al. (2021) [30]. This positions BFRT as a highly effective method for improving mobility and reducing injury risks in older adults.

#### 4.4.2. Safety and Practical Applications

The findings underscore BFRT’s safety and applicability for diverse populations, including older adults and those with limited exercise capacities. For instance, studies by Ozaki et al. (2011) [27] and Shimizu et al. (2016) [25] confirm BFRT’s ability to enhance muscle performance without adverse effects, making it suitable for rehabilitation and fitness contexts.

## 5. Discussion

The present study aimed to review and synthesize clinical evidence regarding the use of blood flow restriction training interventions in healthy older adults, with a focus on identifying effective protocols and optimal parameters. This scoping review analyzed 13 studies evaluating the efficacy of BFRT in this population. The findings indicate that low-load BFRT (20–40% of one-repetition maximum), performed 2 to 4 times per week over a period of 6 to 12 weeks at pressures ranging from 50% to 80% of arterial occlusion pressure, leads to significant improvements in muscle strength (e.g., 15–35% increase in leg press 1RM), muscle hypertrophy (e.g., 5–8% increase in quadriceps cross-sectional area), and functional performance (e.g., 9–21% improvement in Timed Up and Go [TUG] and 6-min Walk Test [6MWT]). These outcomes were comparable to, or in some cases superior to, those achieved through conventional high-load resistance training, with minimal reported adverse effects. The following sections contextualize these findings within the broader literature and discuss their practical implications for exercise prescription in older adults.

Bigdeli 2020 [29] used C-terminal Agrin Fragment (CAF) level as an indicator of muscle atrophy and Neuromuscular Junction integrity; they also used P3NP biomarker for sarcopenia. They attributed the improvement to increased recruitment of fast-twitch muscle fibers during BFR-induced muscle contractions because they induce ischemia and increased metabolite accumulation in muscle fibers, which can activate the afferent nerves III and IV muscle fibers [31]. Also, the larger improvement in the functional training combined with BFR group vs. functional training group may be due to the recruitment of type II fibers, which are more susceptible to atrophy with aging [32]. According to most researchers, BFR training induces adaptations through mechanisms like cell swelling and metabolite accumulation, which activate pathways that stimulate muscle growth. Clarkson et al., 2017 [26] stated that the increased 6MWT distance that the BFRW group was able to achieve is probably due in part to enhanced aerobic capacity (e.g., VO2max). Also, the improvement in TUG might be partially explained by the fact that blood flow restriction exercise recruits more fast-twitch muscle fibers at a lower intensity than higher-intensity options [33]. Cook et al., 2017 [24] noticed that MVC strength increased by 10% in the BFR group between weeks 6 and 12 and attributed the reason for these delayed gains to the fact that in BFR training, strength and hypertrophy adaptations are inverted, delaying the initiation of neural gains that are usually noticeable at the beginning of HL training, as proposed by Loenneke et al., 2012 [33]. Also, it is possible that using a low-load endurance program was better tailored to the BFR group’s training and produced adaptations that were distinct from the HL group.

Karabulut et al., 2010 [19] proposed that the lower initial strength level of older men may be the cause of their greater strength increase when compared to younger men, leading to increased improvements in strength. Neuromuscular adaptations, such as enhanced muscular and/or nerve coordination and the recruitment of additional fast-twitch fibers and their higher threshold motor units, may be responsible for these improvements in skeletal muscle strength [34]. Kargaran et al., 2021 [30] noticed that both the Dynamic Training combined with blood flow restriction (DTBFR) and the Dynamic Training (DT) groups showed a decrease in CAF levels, although the DTBFR group showed a noticeably larger decrease (28.9%) than the DT group (9.2%). CAF may be a biomarker for neuromuscular remodeling and sarcopenia. Also, the 15% improvement in the TUG test seen in the DTBFR group may be explained by this rise in muscle quality and muscular strength.

In Libardi et al.’s 2015 [9] study, the increase in muscle strength might be because fatigue in blood flow restriction combined with resistance training (BFR-RT) can enhance the recruitment of muscle fibers, especially type II fibers [35,36]. Also, the recruitment of the fast-twitch fibers was brought on by adding blood flow restriction to low-intensity resistance training [35]. Increased muscle strength might be mainly due to neural adaptations, according to Ozaki et al., 2011 [28]. Libardi et al. (2015) [9] attributed strength gains to enhanced protein synthesis triggered by BFR exercise [37], with these anabolic responses likely contributing to muscle growth and functional adaptations observed during walking protocols.

There was increase in the muscle strength in a study by Shimizu et al., 2016 [25] but the groups did not significantly differ from one another, and they thought that this increase was because of the acidemia triggered by the high-intensity exercise training, which was observed to enhance the secretion of growth hormone (GH), which in turn contributes to muscle growth [38,39,40], which was also observed in their study. Acidemia enhances neuromuscular adaptation, leading to greater muscle strength [41].

Vechin et al., 2015 [21] demonstrated the reduced response of low resistance training (LRT-BFR) when compared to high resistance training. HRT might be partially attributed to the lower level of neural adaptation shown following this training technique. Also, LRT (i.e., 20–30% of one-repetition maximum) combined with partial BFR (50% of the maximum tibial arterial pressure) provides an effective alternative to HRT for increasing muscle strength and mass in elderly individuals. In Yasuda et al.’s 2016 [22] study, low-load, elastic band resistance training with BFR increased the maximal muscle strength and cross-sectional area in quadriceps but the participants were only older women, so the effect on men is uncertain.

According to (Yasuda et al., 2014) [23], the increase in muscle protein synthesis after exercise seems to be similar between high-intensity resistance training (HI-RT) and low-intensity resistance exercise (LI-RT) with blood flow restriction. Also, the extent of enhancement in chair-stand performance was linked to the increase in muscular cross-sectional area and strength in both the gluteus and quadricep muscles. In (Nielsen et al., 2012)’s [42] study, they showed that blood flow restriction resistance training (BFR-RT) over 23 sessions significantly promotes the growth of myogenic stem cells, leads to the addition of some myonuclei in skeletal muscle, and results in considerable hypertrophy of myofiber.

In a review by Grønfeldt, B. M et al., 2020 [11] that included 16 RCTs, the authors concluded that low-load blood flow restriction (LL-BFR) training and high-intensity strength training both seem to be equally effective at increasing maximal muscular strength in healthy elderly individuals. Therefore, LL-BFR training offers a practical and efficient substitute or addition to traditional heavy-load resistance training. These results coincide with ours [11]. Gao, Z. et al., 2025 [43] concluded that aerobic training combined with BFR significantly increases maximal strength and improves muscle mass. However, the limited effects on muscle size might be attributed to the constraints of low-intensity aerobic exercise in stimulating muscle growth. Wang, T. et al., 2025 [44] in their meta-analysis revealed that both LL-BFR and high-load resistance (HLR) training achieved comparable increases in maximal strength, though LL-BFR resulted in slightly smaller gains; these results also coincide with our study.

While prior meta-analyses (Grønfeldt et al., 2020; Gao et al., 2025; Wang et al., 2025) [11,43,45] established the equivalence of low-load BFRT and high-load training for strength and hypertrophy, this scoping review extends these findings in three key ways. First, we specifically focus on elderly populations, a demographic underrepresented in earlier reviews, and demonstrate that BFRT’s safety and efficacy are retained even in older adults with age-related sarcopenia. Second, we provide insights into protocol standardization (e.g., cuff pressure progression, optimal frequency/duration) tailored to older adults, addressing a gap in prior syntheses. Third, we highlight BFRT’s unique benefits for functional outcomes (e.g., TUG, 6MWT), which are critical for maintaining independence in aging populations. These distinctions position BFRT not merely as an alternative to traditional training but as a prioritized intervention for geriatric populations requiring low mechanical stress.

Many of the studies we reviewed used RPE to adjust cuff pressure and monitor how hard participants were working. While RPE is simple and does not require advanced equipment, we need to be cautious about relying on RPE too heavily for older adults. As people age, their ability to sense their own body’s signals—like fatigue or discomfort—can change. For example, people with mild memory issues might not accurately report how hard they are working. They could downplay their effort to avoid seeming “weak,” or they might push too hard without realizing it, risking injury. This does not mean RPE is useless—it is still a helpful tool. But to make BFRT safer and more effective for older adults, pairing RPE with objective checks could work better.

Practical implications: The most effective BFRT protocols for older adults typically use cuff pressures at 50–80% of arterial occlusion pressure (AOP) for the lower body and 30–50% above systolic blood pressure for the upper body. Wider cuffs (10–18 cm) are commonly used for the lower limbs to achieve occlusion at lower pressures, improving comfort and safety, while narrower cuffs (5–10 cm) are used for the upper limbs. Cuff placement is consistently at the proximal parts of the limb (upper thigh or upper arm). Training sessions are generally performed 2–4 times per week, with each session lasting 10–20 min under cuff inflation. Intervention durations typically range from 6 to 12 weeks. Exercise intensities are maintained at 20–40% of one-repetition maximum for resistance training or about 45% of heart rate reserve for walking protocols. Pressure is often progressively increased by 10–20 mmHg weekly based on perceived exertion ratings, and cuffs are deflated between exercises to prioritize safety. These standardized parameters optimize muscle strength, hypertrophy, and functional gains while minimizing risks in healthy older adults.

Monitoring and Safety Practices: Several studies incorporated rate of perceived exertion monitoring to adjust pressure and intensity dynamically, as in Bigdeli et al., 2020 [29] and Karabulut et al., 2010 [19]. Cuff deflation between sets or after sessions, as practiced in studies like Clarkson et al., 2017 [26], minimized potential risks such as thrombosis or excessive ischemic stress. These safety measures are critical given the cardiovascular and musculoskeletal vulnerabilities common among older adults.

## 6. Limitations and Future Directions

While the findings are promising, some variability in blood flow restriction protocols, such as cuff width, pressure determination, and exercise modalities, underscores the need for standardization to ensure reproducibility and optimize outcomes. Future studies should aim to establish evidence-based guidelines for cuff pressure, placement, and progression tailored to specific populations. While our search strategy excluded studies involving clinical populations (e.g., knee osteoarthritis), this ensured a focused synthesis on BFRT’s efficacy in healthy aging [12]. However, future reviews could explore BFRT’s role in populations with mild, age-related comorbidities to broaden applicability. Additionally, the long-term effects of BFRT on muscle quality and functional independence, particularly in older adults, warrant further exploration.

## 7. Conclusions

Blood flow restriction training is a highly effective, low-risk alternative to traditional strength training methods, offering significant benefits in muscle strength, hypertrophy, and functional performance. Its versatility and safety make it an excellent option for older adults and individuals with physical limitations. To standardize BFRT protocols and maximize their effectiveness, future studies should adopt consistent methods for pressure determination, such as percentage of AOP or systolic blood pressure. Wider cuffs may be preferred for larger limbs, while narrower cuffs can be used for smaller limbs to optimize occlusion and participant comfort. A gradual progression of pressure and intensity is recommended to prevent discomfort and ensure adherence.

Additionally, combining resistance exercises with functional tasks, such as walking with cognitive challenges, could enhance outcomes by addressing multiple aspects of physical function. By standardizing these parameters, BFRT can be further optimized to provide safe and effective interventions, particularly for populations with limited capacity for high-intensity exercise.

## Supplementary Materials

The following supporting information can be downloaded at: https://www.mdpi.com/article/10.3390/jcm14124185/s1, Table S1: Summary of different BFR protocols and parameters; Table S2: Summary of Studies with population characteristics and protocols; Table S3: Summary of muscle strength, hypertrophy, and functional ability outcomes; Table S4: Summary of outcomes/Conclusion.
