# Supplementary material for: Exploring Blood Flow Restriction Exercise Protocols for Elderly Populations: A Scoping Review of Cuff Pressure, Frequency, and Duration for Muscle Strength, Hypertrophy, and Functional Abilities Outcomes
Source: J Clin Med. 2025 Jun 12;14(12):4185. doi: 10.3390/jcm14124185 (PMC12193843; doi:10.3390/jcm14124185)
Supplement: Supplementary file 1 [file jcm-14-04185-s001.zip › jcm-3667946-supplementary.pdf]

Table S1: Summary of different BFR protocols and parameters

ID	Study	Exercise mode<br>Duration/frequency	Cuff / BFRT parameters
1	Bigdeli et al. 2020	Warm-up (10 mins) + functional training (Dumbbell fly, Triceps extension, Shoulder press, Biceps curl, Seated row with power bands on Bosu and Leg curl)<br>6 wk; 3 days/wk	The cuff pressure during exercise sessions started at 50% of the calculated arterial occlusion pressure (AOP) (210-250 mmHg for lower body, 105-130 mmHg for upper body) in weeks 1-2, elevated to 60% in weeks 3-4, and progressed to 70% in weeks 5-6. The rate of perceived exertion (RPE) was used to monitor the intensity of training.
2	Clarkson et al. 2017	Both BFR walking and CON performed 10 min walking (4kmh-¹) around a predetermined path (667 m) in a field setting. The chosen speed and duration selected are representative of common speeds and durations employed in other BFRW research<br>6 wk; 4 days/wk	The BFR cuffs (10.5 cm wide; Dual Port, single bladder cuff) were inflated immediately before the training sessions, and pressure was released immediately following session completion
3	Karabulut et al. 2010	Leg press<br>Leg extension<br>6 wk; 3 days/wk	Placement: Proximal thigh System: Electronic (KAATSU-Master) Pressure: Avg. 205 mmHg, max 240 mmHg Adjustment: +20 mmHg if RPE < 16; same if RPE 16–19 Protocol: 1 min rest (cuffs on), 5–10 min rest (cuffs off), < 10 min inflation/session
4	Kargaran et al. 2021	20-minute walking on a treadmill while executing cognitive tasks<br>8 wk; 3 days/wk	Placement: Cuffs (5 cm width, Ghamat Pooyan, Tehran, Iran) were positioned on the proximal part of the legs. Duration: Cuff pressure was maintained continuously for 20 minutes of walking to ensure a higher metabolic load.<br>Training Pressure: Initial pressure: 50% of calculated AOP, averaging 150–155 mmHg. Progression: elevated by 10% every two weeks, reaching 200 mmHg in the final two weeks.
5	Kim et al. 2017	isometric handgrip contractions<br>4 wk; 3 days/wk	Cuff Type: Straight segmental cuffs SD 10, 11 × 85 cm (D.E. Hokanson, Inc., Bellevue, WA). Placement: The cuff was placed on the upper arm. Cuff Inflation: Inflated to 30% above the subject's systolic blood pressure. Young Participants: Av. pressure of 150 mmHg. Older Participants: Av. pressure of 160 mmHg.

Continued on next page

Table S1: Summary of different BFR protocols and parameters (Continued)

ID	Study	Exercise mode<br>Duration/frequency	Cuff / BFRT parameters
6	Vechin et al. 2015	Leg press<br>12 wk; 2 days/wk	Cuff: 18 cm wide, positioned at proximal thigh. Inflation: Used vascular Doppler (DV-600, Marted, São Paulo, Brazil) on tibial artery; inflated until pulse absent, then released until pulse detected to determine systolic pressure. Pressure: Maintained at 50% of max tibial pressure, adjusted weekly; avg. 71 ± 9 mmHg. Session: Cuff remained inflated throughout.
7	Yasuda et al. 2016	Squats<br>Knee extension<br>12 wk; 2 days/wk	Cuff: 50 mm pneumatic (KAATSU Master, KAATSU Japan), placed at proximal thighs. Pressure: Started at 50 mmHg on Day 1, elevated by 10–20 mmHg per session, reaching up to 200 mmHg as tolerated. Mean Pressure: Averaged 161 ± 12 mmHg, ranging 160–200 mmHg by session 24. Duration: Inflated 10–11 mins per session; deflated after exercises.
8	Yasuda et al. 2014	Knee extension<br>Leg press<br>12 wk; 2 days/wk	Cuff: Elastic pressure cuff, 50 mm wide (KAATSU Master, Sato Sports Plaza, Tokyo). Placement: Proximal part of both legs. Pressure Adjustment: Started at 120 mmHg, increased by 10–20 mmHg each session, reaching up to 270 mmHg. Pressure Selection: Duration: 11 mins per session under moderate restriction. Post-Exercise: Cuff removed immediately after exercises.
9	Cook et al. 2017	Leg curl<br>Leg extension<br>Leg press<br>12 wk; 2 days/wk	Cuff: Pneumatic tourniquet, 6 × 83 cm. Placement: Proximal portion of the leg. Pressure: 1.5× brachial systolic BP, averaging 184 ± 25 mmHg. Timing: Inflated before exercises, released for 3-min rest between sets. Progression: Load increased by 2–5 kg if >30 reps achieved in at least two sets.
10	Libardi et al. 2015	Leg press<br>12 wk; 2 days/wk	Cuff: Standard blood-pressure cuff (175 mm width × 920 mm length) placed around quadriceps (inguinal fold region). Pressure: Inflated until tibial artery pulse eliminated, then set at 50% of this pressure for partial blood flow restriction. Average Pressure: 67 ± 8.0 mmHg during training.

Continued on next page

Table S1: Summary of different BFR protocols and parameters (Continued)

ID	Study	Exercise mode<br>Duration/frequency	Cuff / BFRT parameters
11	Ozaki et al. 2010	Treadmill walking (20 min)<br>10 wk; 4 days/wk	Cuff Placement: 5 cm elastic cuffs on the upper legs. (Kaatsu-Master system)<br>Pressure Increments: Starting at 120 mmHg, increasing by 20 mmHg with 30-second holds, until target pressure.<br>Progression: Day 1 at 140 mmHg, with weekly 10 mmHg increases up to 200 mmHg; only five reached 160–180 mmHg.<br>Release: Cuffs deflated immediately post-exercise.
12	Shimizu et al. 2016	Leg extension Leg press<br>Rowing<br>Chest press<br>12 week; 2 days/week	Lower Body Training:<br>Cuff Placement: 10 cm cuffs on proximal thighs, inflated to femoral systolic blood pressure using a Tourniquet 9000 inflator (VBM Medizintechnik GmbH). 1-minute rest before starting Upper Body Training:<br>Cuff Placement: 7 cm cuffs on upper arms, inflated to brachial systolic blood pressure.<br>Cuff Inflation: Cuffs stayed inflated throughout exercises, deflating only during rest.

Table S2: Summary of Studies with population characteristics and protocols

ID	Study	Population	Protocol, N
1	Bigdeli et al. 2020	Older Men (> 65 y)	FT BFR:25-35% RM (10)<br>FT:Functional training (10)<br>CON: Sedentary life style (10)
2	Clarkson et al. 2017	Elderly individuals (60–80 y)	BFR walking (4 km/h) (10)<br>CON walking (4 km/h) (10)
3	Karabulut et al. 2010	Older males (50–64 y)	LL-BFR (20% 1RM) (13)<br>HL (80% 1RM) (13)<br>CON : sedentary lifestyle (13)
4	Kargaran et al. 2021	Older Women (> 60 y)	DTBFR:40 % of perceived maximal effort (8)<br>Dual-task (DT): Dual training (8)<br>CON: Sedentary lifestyle (10)
5	Kim et al. 2017	Elderly individuals (60–80 y)	BFR: 20% maximal voluntary contraction (MVC) (9)<br>HRT: 75% MVC (10)
6	Vechin et al. 2015	Elderly individuals (59–71 y)	LL-BFR (20–30% 1RM) (8)<br>HL (70–80% 1RM) (8)<br>CG: sedentary life (7)
7	Yasuda et al. 2016	Older females (61–86 y)	LI-BFR : 30% 1RM (10)<br>MH-Tr: middle to high-intensity elastic band training (10)<br>CON: no training (10)
8	Yasuda et al. 2014	Older females (61–86 y)	BFR-RT: 20-30% RM (9)<br>CON: no training (10)
9	Cook et al. 2017	Elderly individuals (65 y)	LL-BFR (30–50% 1RM) (12)<br>HL (70% 1RM) (12)<br>CON: light upper body resistance and flexibility training (12)
10	Libardi et al. 2015	Elderly individuals (> 60 y)	LL-BFR (20–30%1RM) (10)<br>HL (70–80%1RM) (8)<br>CON: no training (7)
11	Ozaki et al. 2010	Older females (57–73 y)	BFR walking (45% HRR) (10)<br>CON walking (45% HRR) (8)
12	Ozaki et al. 2010	Older females (57–76 y)	BFR walking G(45% HRR)(13)<br>CON walking G(45% HRR)(10)
13	Shimizu et al. 2016	Elderly individuals (> 65 y)	LL (20% 1RM) (20)<br>LL-BFR (20% 1RM) (20)

Abbreviations: FTBFR = Functional training Blood Flow Restriction ; HRT = High resistance training ; DTBFR =Dynamic Training Blood Flow Restriction ; LRT = Low Resistance Training; ES = Effect Size ; LI = Low Intensity ; MH-Tr = middle to high intensity training ; CON = Control

Table S3: Summary of muscle strength, hypertrophy, and functional ability outcomes

ID	Study	Muscle strength	Hypertrophy	Functional ability
1	Bigdeli et al. 2020	Knee Extension (kg) FTBFR: 18.6% d = 2.2 FT: 15.5% d = 2.1 Control (C): 5.8% d = -0.4 Chest Press (kg) FTBFR: 20.4% d = 1.9 FT: 16.6% d = 1.6 Control (C): 9.5% d = -1.1		Modified Romberg Test (s) FTBFR: 43.6% FT: 54.3% Control (C): 5.0% Timed Up and Go Test (s) FTBFR: 21.0% (indicating a decrease in time, which is an improvement) FT: 28.4% (also indicating a decrease in time and an improvement) Control (C): 1.8% (indicating no meaningful change)
2	Clarkson et al. 2017			30-Second Stand-Up Test(%) BFR: 28% CON: 8% TUG (%) BFR: 12% CON:5% 6MWT (%) BFR: 9% CON:2%
3	Karabulut et al. 2010	Leg press (Kg) LL-BFR: 19.3% d= 1.7 HL: 20.4% d= 1.86 Knee extension(Kg) LL-BFR: 19.1% d= 1.62 HL: 31.2% d= 2.56		
4	Kargaran et al. 2021	30-Second Stand-Up Test (N) DTBFR: 19.3% increase, d = 1.9 DT: 0% change, d = 0 Control (C): -3.3% (decrease), d = -0.4 Biceps Curl (kg) DTBFR: 4.8% increase, d = 2.1 DT: 4.3% increase, d = 1.1 Control (C): 1.3% increase, d = 0.7		Timed Up and Go Test (s) DTBFR: -15.6% (decrease in time, improvement), d = -1.4 DT: -4.2% (decrease in time, improvement), d = -0.8 Control (C): 2.6% (increase in time, no improvement), d = 0.4 Sharpened Romberg Test (s) DTBFR: 23.1% increase, Cohen's d = 0.8 DT: 5.4% increase, d = 0.6 Control (C): 0% change, d = -0.1 6-Minute Walking Test (m) DTBFR: 6.4% increase, d = 1.5 DT: 2.5% increase, d = 0.5 Control (C): -1.2% (decrease), d = -0.4
5	Kim et al. 2017	Strength (kg): BFR: +8.7% increase, d = 0.44 HRT: +17.6% increase, d = 1.27	Girth (cm): BFR: +1.5% increase, d = 0.36 HRT: +0.8% increase, d = 0.19	
6	Vechin et al. 2015	Leg Press 1RM HRT : 50.3% increase ES = 1.50 LRT-BFR : 15.8% increase ES = 0.59 CON: -9.4% decrease ES Not significant (p = 0.998)	Quadriceps Cross-Sectional Area (CSA) HRT: 7.4% increase LRT-BFR: 5.9% increase CON: -1.7% decrease	

Continued on next page

Table S3: Summary of muscle strength, hypertrophy, and functional ability outcomes (Continued)

ID	Study	Muscle strength	Hypertrophy	Functional ability
7	Yasuda et al. 2016	Knee extension MVIC Knee extension LI-BFR :13.7% MH-Tr: no improv CON: no improv Leg press 1RM LI-BFR : 16.4% increase MH-Tr: 17.6% increase CON : no improv	Quadriceps Cross-Sectional Area (CSA) LI-BFR : 6.9% increase MH-Tr: 1.5% increase CON: -2.2% decrease	
8	Yasuda et al. 2014	Knee extension: BFR-RT: 26.1% CON: no improv Leg press: BFR-RT: 33.4 CON: no improv	Quadriceps CSA BFR-RT:8% CON: -1.8%	Chair stand test: BFR-RT: 18% CON: 2.2%
9	Cook et al. 2017	Knee extension : BFR: 24% HRT: 26% CON: no imporov	Quadriceps CSA BFR: 4.3 % HRT: 3.6 % CON: no improv	SPPB: BFR: MD = 0.09 HRT: MD = 0.02 CON: MD = 0.44
10	Libardi et al. 2015	1 RM : LL BFR: 35.4% HRT: 38.1% CON: No improv	Quadriceps CSA : LL BFR: 7.6 % HRT: 7.3 % CON: no improv	
11	Ozaki et al. 2010	Muscle strength BFR: 3–22% CON: 4 to 2%	Mid thigh CSA BFR: 3.1 % CON: 0.1	
12	Ozaki et al. 2010	Muscle strength BFR: 9-15% CON: 0-3 %	Thigh CSA BFR: 3.2 % CON: -0.2	
13	Shimizu et al. 2016	Muscle strength LL: 2 to 7% LL-BFR: 6–19%		

Table S4: Summary of outcomes/ Conclusions

ID	Study	Outcomes/ Conclusion
1	Bigdeli et al. 2020	Both FTBFR and FT groups show significant improvements in strength, balance, and mobility (Cohen's d > 1.5), while the control group exhibits no improvement or slight declines.
2	Clarkson et al. 2017	Significantly greater enhancement in capability across all functional mobility assessments for BFR group versus CON within this elderly population.
3	Karabulut et al. 2010	No significant variations between group with the exception of dynamic leg extension (superior in HL) Cohen'd= 2.56 but leg muscle strength improves with both training protocols when compared with CON in older men
4	Kargaran et al. 2021	DTBFR Group: Significant gains in strength, balance, and performance with large effect sizes, highlighting DTBFR's effectiveness.<br>Dual-Task (DT) Group: Moderate improvements in strength and mobility with smaller effect sizes, less impactful than DTBFR.<br>Control (CON) Group: Minimal or negative changes, underscoring the need for active interventions to enhance performance.
5	Kim et al. 2017	Both BFR and HRT show significant improvements in strength, with O-HI demonstrating a much larger effect size. In terms of girth, BFR shows a more noticeable improvement, though the effect size for girth changes in both groups is smaller.
6	Vechin et al. 2015	Both HRT and LRT-BFR groups showed substantial improvements in strength (leg press 1RM) and muscle size (quadriceps CSA), with HRT yielding a larger effect size for strength gains. The Control Group displayed no meaningful changes, supporting the efficacy of resistance training, especially HRT, in enhancing these physical outcomes.
7	Yasuda et al. 2016	The LI-BFR group achieved the most improvement in strength and muscle size, followed by MH-Tr, which showed moderate strength gains. The Control group showed no improvements, reinforcing LI-BFR's effectiveness.
8	Yasuda et al. 2014	The BFR-RT group demonstrated significant improvements in both knee extension and leg press strength, as well as an increase in quadriceps muscle size (CSA) and functional ability. In contrast, the Control group showed no improvements in strength measures or functional ability and a slight decrease in quadriceps muscle size.
9	Cook et al. 2017	The overall enhancements indicated that HL training produced notably superior increases in muscular strength 1-RM and CSA than the CON group. The BFR training group acquired significantly greater muscular mass than HRT and CON. Also, none of the interventions led to a reliable improvement in the 400-m walk speed across these groups.
10	Libardi et al. 2015	Both Strength, and muscular hypertrophy improved to a similar extent when endurance training (ET) was combined with either high-intensity resistance training (HI-RT, traditional concurrent training) or (BFR-RT, BFR-CT). These results indicate that BRF-CT could serve as a viable alternative to current exercise guidelines for Elderly population.
11	Ozaki et al. 2010	Significant improvement in muscle strength and CSA in mid thigh for BFR group
12	Shimizu et al. 2016	No significant betweengroup differences
